# Governing Through Ignorance: Swedish Authorities’ Treatment of Detained and Non-deported Migrants during the COVID-19 Pandemic

**DOI:** 10.1007/s10691-022-09495-5

**Published:** 2022-09-17

**Authors:** Annika Lindberg, Anna Lundberg, Elisabet Rundqvist, Sofia Häythiö

**Affiliations:** 1 The School of Global Studies, University of Gothenburg, Konstepidemins väg 2B, 413 14 Göteborg, Sweden; 2 Sociology of Law Department, Lund University, Box 42, 221 00 Lund, Sweden; 3 The Swedish Network of Refugee Support Groups (FARR), Box 391, 101 27 Stockholm, Sweden

**Keywords:** COVID-19, Deportation, Ignorance, Migration-related detention, Sweden

## Abstract

Tensions between migration enforcement and migrants’ health and rights have gained renewed urgency during the COVID-19 pandemic. This article critically analyses how the pandemic has affected detained and deportable people in Sweden. Building on an activist methodological approach and collaboration, based on a survey conducted inside Swedish detention centres during the pandemic and the authors’ research and activist engagement with migrants who are detained or legally stranded in Sweden, we argue that migration authorities’ inadequate measures to protect detained and deportable people during the pandemic is a case of governance through ignorance enabled by structural racism. The article traces how this ignorance operates on a structural, institutional and micro-level, enabling public disregard and political irresponsibility for the harmful effects of migration enforcement. A broader aim of the article is to challenge the structural, societal and epistemic ignorance of the conditions for detained and deportable persons and to contribute to political change.

## Introduction

Previous feminist studies have highlighted that oppressed groups have been particularly hard hit during the pandemic (see Bowleg 2020; Esposito et al. 2021). COVID-19 has brought into light the pre-existing unevenly distributed vulnerabilities among different communities, which are structured along the lines of class, race and gender—and legal status. In the Swedish context, cramped housing conditions, gender-based violence, the need to travel by public transport and forms of work which do not enable working from home, for example among care professionals, have been highlighted as examples of such vulnerabilities (see for example Rambaree and Nässén 2020; Socialstyrelsen 2020; Harris and Sandal 2021; Jämställdhetsmyndigheten 2021).

Among those most harshly affected are migrants who lack legal authorisation to remain, and who because of increasingly restrictive migration policies are excluded from social protection systems, and exposed to destitution, detention, and deportation. In some respects, the pandemic caused a rupture to migration control enforcement, since much international travel, including deportations, was decelerated, or stopped altogether. Yet, most states have continued detaining and deporting people where possible, disregarding the health hazards of international travel for deported individuals—and the risk of the virus spreading further in the countries that they were deported to. While some countries released non-deported migrants from detention and temporarily granted them access to minimum social welfare during the pandemic (see Roman 2020), most states continued to prioritise deportation enforcement over migrants’ health. While this is not a novelty, the pandemic re-actualised issues of access to health, to basic social rights, and indeed, to life, for deportable people.

This is also the case in Sweden, which is the empirical focus of the present article. During the pandemic, migration authorities continued to detain, deport, and render non-deported migrants destitute. Meanwhile, the effects of the pandemic on the legal and social condition of this group have been entirely absent from Swedish political and public debate, despite protests organised by detained migrants and efforts by civil society actors and researchers, including the authors of this article, to draw attention to their situation. Rather than considering this a mere failure on behalf of migration authorities to protect their lives and to implement official guidelines and regulations, we propose to understand the lack of consideration for non-deported migrants’ health and safety during the pandemic as a form of governance through *ignorance,* which operates on several levels, and which can be understood through the racism underpinning border and migration politics in Sweden.

Drawing on critical border research and agnotology studies, we explore how ignorance as a governing strategy, which is rooted in racialised perceptions of whose lives are worth safeguarding, and whose lives are disposable (Gilmore 2007), enables neglect of the harms caused by law enforcement to racialised ‘Others’ (Gross and McGoey 2015). Empirically, the article builds on the findings from a survey undertaken as a collaboration between volunteers in the Swedish Network of Refugee Support Groups (FARR) and researchers, within the framework of the Asylum Commission (Häythiö et al., 2020). The purpose of the survey was to investigate the extent to which Swedish migration authorities had taken measures to protect detained foreign nationals during the pandemic. A digital questionnaire was distributed among people held in Swedish detention centres in spring 2020. In addition to the survey results, the article builds on knowledge generated through interviews with people who sought protection in Sweden, social workers, and non-governmental organisations (NGOs) supporting non-deported persons, conducted between 2015 and 2020, and text analysis of policy and legal documents. It is also informed by the authors’ activist and professional work with people living under threat of deportation.

Hence, using the expert witness testimonies of people who are detained or live in a precarious condition of deportability, we analyse Swedish migration authorities’ (non)response to the pandemic and how it has affected those who are detained or live under threat of deportation. While the empirical focus of the article is on (pre-deportation) detention during the COVID-19 pandemic, the case enables us to discuss the broader structural and institutional disregard for the ineffectiveness and harmful implications of deportation enforcement. A broader ambition of the article is thus to challenge political and public invisibilisation of the conditions facing detained and deportable persons, and to contribute to progressive political change.

In what follows, we elaborate on the theoretical framework of the article, where we build on critical, feminist and socio-legal scholarship on migration and deportation as well as ignorance studies. We then describe our survey design, including its limitations, and our methodological transdisciplinary approach. The results of the study are presented in three sections, where we depart from the observations made by people held in Swedish detention centres during COVID-19 and our prior research. In the discussion section, we point to the role and effects of governance through ignorance in detention and deportation processes and how it impacts non-deported people. We further reflect on the implications of ignorance for society at large.

## Contextualising the Research: The Governance of Non-deported People

The study focuses on the conditions facing people threatened by deportation in Sweden during the COVID-19 pandemic. Deportation is a technique of statecraft: it is a tool through which states manage populations and assert control over bodies and territories (Peutz and De Genova 2010). It is also a differentiation device: critical border scholars have pointed out how migration and deportation regimes rely on and produce racial hierarchisations of human life (Davies et al. 2017; Mayblin et al. 2019). In addition to racialisation (Ellermann 2020; Khosravi 2020), migration/deportation laws are structured along the lines of ascribed group membership such as gender, class and sexuality (Askola 2010; Esposito et al. 2020a, 2020b). Vulnerability to migration enforcement, including detention, deportation, and associated harms, is distributed accordingly. Meanwhile, critical feminist literature on borders has highlighted how gender-specific vulnerabilities are often ignored altogether in processes of migration control enforcement (Sager 2016; Canning 2019). Or, such vulnerabilities are reduced to monolithic classifications, where migrant women are portrayed as ‘victims’ (of trafficking and other gendered exploitations; see Askola 2010) and male migrants are treated as threats, imbued with danger, illegality, and criminality, and hence denied vulnerability (Wyss 2022). Racialised, gendered notions of threat and vulnerability are also reflected in the deportation process, crucially including migration-related detention.

Migration-related detention centres are, on the one hand, hypervisible, as ‘spectacular' mechanisms of state control over mobility (De Genova 2013). On the other hand, their daily operation is obscured from public view—and to a large extent, from external scrutiny. Detained migrants find themselves at the margins (Eule et al. 2019) of migration control bureaucracy in a temporal, physical and legal sense. Prior research has demonstrated how detention is a racialised and gendered state practice: exposure to detention and deportation is unevenly distributed according to nationality, which serves as a proxy for ‘race’ (Mongia 2018). The majority of those who are detained in Sweden and in most other countries are men (Griffiths 2015; Bosworth 2019), who are represented as undeserving, potentially criminal, and disposable (Khosravi 2020). The nuances of gendered experiences of detention, including the specific gendered subjectivities that are constructed through detention and detainability, have been analysed in-depth by feminist border scholars (see Askola 2010; Griffiths 2015; Esposito et al. 2020a, 2020b).

In this article, we are concerned with one particular effect of the gendered and racialised practice of detention: namely, the construction of detained people as undeserving subjects, who can (legitimately) be denied care and protection and who are thus, in the context of a global pandemic, *made* vulnerable to state (in)actions (Canning 2019). Departing from the anonymous testimonies of people detained during COVID-19, we explore what their shared experience of vulnerability and of state abandonment can tell us about deportation regimes, and of the racialised boundaries of the ‘caring’ Swedish welfare state.

We use *ignorance* as a theoretical concept to analyse the systemic disregard for detained and non-deported people’s health and well-being during an ongoing pandemic. Ignorance can be understood as a state of not-knowing, lacking wisdom/judgment, or of being consciously unaware of something. As Cohen (2001) argues, however, ignorance is not only a psychological mechanism but also a social and political force. Within the field of ignorance studies, scholars have shown how the production of “absences of knowledge” (Croissant 2014, 11; Borrelli 2018) can be instrumentalised for the purpose of governance (Gross and McGoey 2015; Stel 2016). Ignorance can, for instance, be imposed by politicians on a population, or feigned for the purpose of deflecting responsibility for adverse outcomes of a particular policy. It may also be maintained on the societal level as to preserve social order and existent inequalities. Paul Gilroy (2006) has emphasised the need to pay attention to the patterned and, indeed, orchestrated denial of colonial histories and their contemporary reverberations. Such denial prevails in the Nordic countries, which falsely position themselves as ‘exceptions’ to these histories, disavowing their complicity and participation in the formation and reproduction of racialised global inequalities (see also Keskinen et al. 2009). This manufactured ignorance obscures how racism—these days often mobilised around cultural and religious traits or national origin, rather than biological features—continues to operate as a device for classifying and hierarchically ordering people considered un-belonging to the welfare state and nation, and for denying them access to fundamental rights and freedoms. Migration laws and policies are among the sites where we can observe the effects of orchestrated ignorance—of the racism underpinning policy, and of its harmful implications for racialised migrants—at work on the political and bureaucratic levels. Indeed, next to coercive control, orchestrated ignorance has been identified as an important governing technique for states’ migration control apparatuses (Kalir and van Schendel 2017; Canning 2018). Ignorance is a key tenet of structural violence, which enables impunity and irresponsibility for avoidable human suffering and unjust outcomes of laws and political decisions—when these concern people who are racialised as migrant ‘Others’.

Law not only ascribes responsibility, but also organises irresponsibility by delineating ‘zones’ where no one is accountable or responsible for conditions that put people at risk or subject them to avoidable harm (Veitch 2007). Our understanding of law (more precisely in our context, detention and deportation law) draws on Susan Coutin, who emphasises how law is “more than legal codes, government policies, and bureaucratic apparatuses” (1993, 88). More than black letter text, law is shaped by various actors, processes and institutions involved in the control of migration. In this article, we trace the actors, processes and practices that partake in and thus enable the orchestrated ignorance of the health, well-being, and lives of racialised detained and deportable people during the pandemic.

## Methodological Considerations

The article is based on a survey study, which was designed in close collaboration between FARR, Anna Lundberg, and Annika Lindberg, in the context of the research initiative the Asylum Commission.^1^ Via their network, members of FARR were able to identify issues regarding authorities’ treatment of detained and deportable persons during the COVID-19 pandemic. From their detention visitor’s group, FARR gathered that many detained people were frightened and experienced that their health was completely ignored by the migration authorities. The methods for addressing their concern and analysis of the results were developed through ongoing exchanges between FARR and researchers working within the Asylum Commission. The present study is therefore the result of collaborative work, which for us has entailed combining experiences with a theoretical understanding, working collaboratively between activists and researchers and—as a stated ambition—transforming the conditions for detained migrants and challenging the deportation politics that incarcerate them. Such collaboration was crucial to be able to carry out the survey inside Swedish detention centres, where external scrutiny is very limited. Through FARR’s voluntary visiting group, contacts were already established with people confined in Swedish detention centres. When the pandemic struck, detained people reported deficient health and safety routines, and widespread fear and anxiety among those incarcerated. Their concerns gave rise to the idea of conducting a survey. It was therefore an important aspect of the work behind this article, and part of the activist methods as we understand them, that we first published the results in a popular scientific report, which early on after the investigation was publicly available and accessible to a wider audience including the press (in English and Swedish). The primary purpose of the research was thus not to publish scientifically reviewed texts, but to work towards political change by drawing public attention to the condition of detained migrants and putting pressure on relevant authorities to protect detained and deportable migrants’ health, rights, and dignity.

The digital survey was distributed among people detained in five of Sweden’s six detention centres in spring 2020, at a time when COVID-19 was steadily spreading in Swedish society. The aim of the survey was to explore if Swedish migration authorities had followed their own recommendations and regulations to safeguard the health of detained people during the pandemic. The survey questions were posed in Swedish and English and supported by images, borrowed from the health and medical care services’ image support, which they use in health conversations with newly arrived immigrants. Questions were asked regarding occupancy in the detention centres, hygiene routines, availability of information, and access to healthcare. It included multiple-choice answers, for example, regarding the availability and usage of protective gear among staff, scales to assess the hygienic conditions in the centre, as well as open questions where respondents were invited to write freely about their experiences.^2^ It was distributed via a digital link that was sent to people confined in different detention centres, whom FARR were already in contact with, and who in turn circulated it among other detained people. Respondents were thus selected via a random snowball sample method. Due to COVID-19-induced restrictions on physical visits to the detention centres, it was not possible to ensure that all detained people received the survey.

Hence, the survey method came with some crucial limitations: we cannot know how many people received the link and given the gender-based segregation of detention facilities, we suspect that most respondents—if not all—were people identifying as men. Language restrictions posed another obstacle, as people with no English or Swedish speaking contacts on the inside were not able to answer. However, given that many of those who are detained have lived in Sweden for a long time, we can assume that those who received the link could also respond to the survey. While personal relationships of mutual trust between members of FARR and our contact persons within detention centres were crucial for the distribution of the survey, we also received indications that some detained people were reluctant to fill out the survey for fear of repercussions if staff would find out what they had reported to us. This is not only important to acknowledge as a limitation to our methods but also shows us the mistrust that detained people feel towards the Swedish migration authorities, which impacts their possibilities to criticise the conditions in detention. Other researchers working on migration-related detention have highlighted the structural obstacles and ethical dilemmas characterising research in these sites, which are fraught by violence, power inequalities and interpretive limitations (Bosworth and Kellezi 2016). Under such conditions, the intent to work with participatory approaches to knowledge production that centre marginalised voices is compromised. Aware of these limitations, we invite scholars and activists to elaborate upon our methodological approach to strive towards re-centring marginalised persons as expert witnesses, and to shed light on the otherwise obscured sites of migration enforcement.

We received 58 responses (representing 36.8% of the detained population at the time of the survey) from five of Sweden’s six migration-related detention centres. Our collaborative approach enabled us to cross-check our findings and analysis with each other and with people directly affected by the migration control policies and practices under scrutiny. Besides the survey, this article is informed by several years of conversations and interviews with people who sought protection in Sweden, and with social workers and NGOs working to support non-deported people in Sweden, conducted between 2015 and 2020. Moreover, we draw on our experience of providing legal advice and information to people who are stuck in limbo, and text analysis of policy and legal documents and state authorities’ internal guidelines.

The main source of knowledge about the conditions for detained and non-deported persons comes from the people targeted by the policies under scrutiny. In using a methodology that builds on the testimonies of people whose voices and life conditions are regularly overlooked in policy and public debates, we are indebted to postcolonial and Black feminist scholars who have highlighted how ignorance of the ‘Other’ is not only political but also epistemic. Patricia Hill Collins (2000) and Boaventura de Sousa Santos (2014) have highlighted how the knowledges of oppressed groups have consistently been disregarded and discredited as a means of exerting power and denying their humanity. Santos (2014) argues that the hegemonic, Western epistemology and the rationality it heralds produces “forms of non-existence” (2014, 118). Gayatri Spivak (1998) uses the term ‘epistemic violence’, and Miranda Fricker (2007) uses ‘epistemic injustice’, to describe the disappearing of the knowledge of marginalised groups, which undermines their ability to speak and to be heard.

This issue also prevails within research dealing with matters of social injustice, including migration studies. People who migrate to seek protection or better life opportunities are routinely portrayed as objects of academic knowledge production but are rarely acknowledged as co-producers of knowledge (Grosfoguel et al. 2015). In other words, even though migrants might *speak* through these accounts, they are rarely considered as *knowers* (Boochani et al. 2020). With this contribution, our hope is to counteract a knowledge deficit that characterises research on migration control practices, even as we, due to our privileged position, are limited in our understanding of the embodied, everyday experiences of the damage caused by immigration legislation, and of the compounded nature of these harms.

This work has been informed by a collaborative *activist methodological approach*, with an explicit transformative aim (see Mobjörk 2010), and with an analytical approach that builds on a unique collaboration between researchers and civil society actors (for other studies with similar approaches, see Martin 2013; Söderman 2019; Wood 2019; Lind 2020). With the ambition to combine theoretical concerns and practical experiences, our activist approach is based on the feminist standpoint that the *experiences* of those who are directly affected by structures and politics, are central to understanding social phenomena (Mulinari and Sandell 1999). Hence, the research approach in the present study is based on the presupposition that detained migrants yield an expert insider knowledge and they are accordingly constructed as the “privileged subject of history” (Napels 2010, 510). This means, in turn, that our collaboration enables knowledge to come to the forefront that would otherwise be impossible to achieve.

Our aim, moreover, is to use our collective knowledge to bring about transformative political change. In their review of how Black feminist approaches and the theory of intersectionality took root in Swedish academia at the turn of the millennium, feminist scholars Paulina de los Reyes and Diana Mulinari (2020) emphasise the explicit ambition of this scholarship to provide *politically* relevant analyses of economies’ effects on human lives in a postcolonial world. Intersectionality also brought an ambition to contest inequalities within (white) academic work. The concept was born out of the Black feminist critique of hegemonic ‘White feminism’, which lacked an understanding of racism. Yet, as de los Reyes and Mulinari (2020) underscore, intersectionality as a political project has since endured a process of academisation and a dissociation of academic knowledge production from everyday struggles. In our work, we wish to emphasise our indebtedness to this body of intellectual work and our commitment to realign our scholarship with the struggles of, in this case, migrants who are marginalised along intersecting axes of oppression. In doing so, we heed the calls by feminist scholars for trans-disciplinary visionary research (see Martinsson and Mulinari 2018; Lundberg 2022).

## Research Findings: How Ignorance Structures Swedish Migration Authorities’ Treatment of Detained and Non-deported People during the COVID-19 Pandemic

In the following analytical section of the article, we trace the different manifestations of governance through ignorance that we find in Swedish migration authorities’ treatment of detained and non-deported migrants during the pandemic. We first introduce the Swedish deportation regime, and account for the concerns raised by detained migrants regarding the everyday, micro-level expressions of ignorance. In a second step, we trace the lack of responsibility for detained migrants’ health and well-being to the institutional and structural level.

### The Swedish Case

The Swedish Aliens Act (2005:716) permits migration authorities to use administrative detention and/or withdraw access to basic welfare provisions for deportable persons.^3^ The Swedish detention regime comprises six migration-related detention centres dispersed throughout the southern and central parts of the country. Detention centres are run by the Swedish Migration Authority (SMA) and have capacity to incarcerate around 500 people. The SMA and the police can make decisions to detain people who lack authorisation to remain in Sweden, to investigate their identity, or to enforce a deportation order (Aliens Act ch. 10–11). Most detained individuals are awaiting deportation. The Migration Agency is bound to run the detention centres in a “purposive and efficient way” (Migrationsverket 2020, 15) and to ensure that detained persons are treated in a “humane and dignified” manner (DeBono et al. 2015, 194). Detaining in a ‘purposive’ manner means that cases where there are reasonable prospects for a deportation order to be enforced should be prioritised. A person can be incarcerated for a maximum period of 12 months and the detention order should be repealed when there are no longer grounds for prolongation (Aliens Act ch. 10 s 9). In practice, however, these notions of ‘humaneness’, efficiency, and rule of law, which also characterise the public imagination of how Swedish migration authorities operate (see for example Feijen and Frennmark 2011), stand in sharp contrast to reports of uneven and arbitrary implementation of laws and regulations and lack of measures to hold authorities accountable.

Prior research on asylum assessments (Lundberg 2011; Johannesson 2017; Bergström et al. 2019), and detention and deportation processes in a Swedish context (Eule et al. 2019; Lindberg 2020) has shown how legal and bureaucratic procedures are often ridden with inconsistencies and arbitrariness (Asylum Commission 2020; Elsrud et al. 2021). Yet the limited monitoring mechanisms (which include the Parliamentary Ombudsman, the Migration Agency’s own Ethics Committee, and internal inquiries), and high trust in government authorities and their compliance with law ‘on paper’, enable disregard for the actual consequences of law enforcement (Lundberg 2011, 2020) and lend legitimacy to government (in)actions (Borrelli and Lindberg 2020, 7). The importance of public trust in government is also reflected in the SMA’s internal governance, where the ideal of loyal public officials acting in accordance with orders from above is strong. It ultimately creates a circular argumentation, where external scrutiny is perceived as superfluous due to the assumption of good performance on behalf of state authorities. Critique voiced by people affected by immigration legislation as well as by external actors (such as the Ombudsman or FARR) is regularly discarded with reference to policymakers’ and managers’ trust in rules and regulations being followed on the street level. As a result, systemic deficiencies can remain ignored for a long time.

These deficiencies became acutely visible during the COVID-19 pandemic. Throughout the duration of the pandemic, the conditions facing people who are detained or pushed into destitution while awaiting deportation have been nearly absent in Swedish political and public debate. While several other European countries took political initiatives —however temporary and limited—to protect these groups during the pandemic, by releasing migrants from detention (Spain, Italy), by ensuring access to healthcare and social rights (Portugal), or suspending certain deportations (Germany), no corresponding political measures were taken in Sweden (see Global Detention Project 2020; Roman 2020). In public debates on Sweden’s strategy to contain the spread of the virus, the conditions of people detained on immigration-related grounds were entirely absent. The only aspect mentioned in media regarding detained and deportable people’s situation was the difficulties facing migration authorities when they kept trying to deport people to countries which refused to accept back their citizens (Larson 2020). The perspectives and experiences of detained and deportable persons have, however, not come to the fore.

On 1st April 2020, the Swedish public health authorities issued recommendations for the Prison and Probation Service, the SMA and the National Board for Institutional Care on how to contain the spread of the virus within their respective institutions. In addition to these new guidelines, the SMA should follow the Communicable Diseases Act (2004:168) as well as internal regulations and action plans for how to contain infectious diseases within their institutions (see Häythiö et al. 2020, 4, for full list of internal regulatory documents). What the effects where, from the perspective of detained people, will be presented below. We organise our findings under three themes: ‘disregarding non-deportability’, ‘ignorance of unattainable regulations’, and ‘indifference to the health and well-being of detained migrants’. Each section begins with excerpts from the responses to the survey, containing testimonies from people incarcerated in the detention centres, whose accounts we have then compared to the adopted rules and regulations for how to handle the pandemic. Finally, we discuss the conditions of non-deported people who have been pushed into destitution, and the lack of measures taken to protect them during the pandemic.

### Disregarding Non-deportability: Detaining during an Ongoing Pandemic

“They bring lots of people from outside at this moment without any medical check, and when we complain they tell us that ‘we have no capacity to check these people', because they know we can’t do anything, no one will listen to our voices.”

“No one is responsible for us, police and Swedish Migration Agency and staff told me, therefore we don’t eat food for six days. Maybe it [the virus] comes from the food or from those who prepare it, nobody knows if they have COVID-19.”

“Sweden has no consciousness, just business of us [sic].”

These testimonies were provided by detained migrants who answered our survey during a period when COVID-19 was rapidly spreading in Swedish society. They highlight how people were detained without medical checks, and how those detained were left with the suspicion that anyone, including staff, other detained people, or food delivery services, could be potential sources of contagion. As the quotes illustrate, respondents reported feeling abandoned and that no one took responsibility for protecting their health.

In this light, the first expression of ignorance that we find in Swedish migration authorities’ (non)response to the pandemic is the fact that police and migration authorities continued to detain migrants during the pandemic, even as flights were cancelled, and countries closed their borders across the world. This practice of continuing deportation enforcement as usual demonstrated a lack of consideration of the fact that we were in a pandemic—a situation requiring comprehensive measures to combat the spread of the infection—*and* of the practical impediments to enforcement due to borders closing and entire societies going into lockdown. The Swedish police authorities, who are responsible for the majority of the administrative decisions to detain migrants in Sweden, reportedly released some 200 people from detention in April 2020 due to the impossibility of enforcing their deportation orders. The reason hereto was, according to the police, that many countries closed their borders to contain the spread of COVID-19; yet the police also vowed that they would continue working ‘hard’ to detect, detain and deport migrants also during the pandemic (Larsson 2020). In doing so, the police authorities demonstrated ignorance of the health risks that deportation entails for both the deported person and the country of deportation. For instance, whereas countries like Afghanistan asked deporting countries to halt deportations due to the virus, in December 2020, Swedish police authorities reported that they would oblige deportable people to take a COVID-19 (polymerase chain reaction, PCR) test to ensure that they would be readmitted to Afghanistan (Engström 2020). The implementation of such a routine has been confirmed by research participants we talked to.

The decisions to continue enforcing deportations were taken at a time when the European Commissioner for Human Rights and the United Nations High Commissioner for Refugees (UNHCR) urged states to immediately release migrants from detention and to the greatest extent possible use alternatives to detention to protect their health and safety during the pandemic (European Commissioner for Human Rights 2020; UNHCR 2020). Swedish authorities’ failure to answer to these calls is not only an indicator of their disregard for migrants’ health, but also demonstrates ignorance of the practical impediments to deportation enforcement. As one respondent put it, Sweden prioritised maintaining the ‘business’ of detainment and deportation over migrants’ lives.

### Ignoring Unattainable Regulations? The Lack of Hygiene Routines Inside Detention Centres

“The biggest risk is that we are infected by staff. There is not much protective gear.”

“We have people here who are infected, and we eat our meals around the same table. One was very ill and when they wanted to take him to the hospital, he died in the hospital. When they arrived there, he had died. We have seen on tv that we need to keep distance from each other, but you can’t do that here because when you eat food there are at least six people around a table. When you want to use the computer to talk to your family, you must sit really close to each other. We have 20 computers in a room that is 4×5 metres, so you can imagine how close we are sitting. Almost everyone is sick, but you can’t get the same help you get on the outside in here because here they don’t take it so seriously.”

“There are no cleaning products at all. When we ask for alcohol or some other disinfectant, they tell us that they don't have enough. Our rooms are not safe (verbatim) [sic] and secure, some of the people even sleep out of the room because this phenomenon threatens their lives.”

From these quotes and the survey responses, which we compared to authorities’ internal rules and regulations, we could identify several shortcomings in hygiene routines inside detention centres. Three quarters of the respondents (74%) reported that staff lacked protective gear; they also reported a lack of hand sanitiser, unsatisfactory cleaning routines, and great difficulties maintaining physical distance during meals and in common areas. The limited possibilities to maintain distance and follow hygiene routines inside detention centres have been highlighted in several reports from migration-related detention centres elsewhere and were among the reasons why states have been encouraged to release migrants from detention (Roman 2020; UNHCR 2020). The survey responses highlighted how this caused severe anxiety and unease among detained migrants. Some of them said they would sleep in the common rooms for fear of becoming infected inside the sleeping rooms, which they shared with other detained people. Detained migrants’ testimonies further stand in sharp contrast to the internal guidelines on the hygiene measures that Swedish migration authorities should adopt to prevent the spread of the virus (see Häythiö et al. 2020).

Detained migrants were also, due to lack of clear communication from authorities, kept ignorant of health-related regulations and recommendations during the pandemic. The survey responses indicated that detained migrants received insufficient information regarding how to protect themselves and others from the virus. 65.5% of the respondents shared that they had received written information to ‘wash their hands and maintain physical distance to others’, while 19% stated that they had not received any information at all. In their written responses, respondents shared the following:

“The staff have not informed me about [the risks of] Corona. They say it’s not dangerous; we are sitting 30–35 people together in a small dining hall and sometimes there is no space where we can sit.”

“Sometimes we are five to six in numbers in one room. I just saw information about the coronavirus on the notice board. We don’t have any social distance in our detention centre. We all go and have lunch, dinner and supper together and we are like six to seven people at one table.”

These testimonies, and the anxiety that they reflect among respondents, is illustrative of the general uncertainty that prevails in migration-related detention, which keeps legal decision-making and practice opaque to migrants and which other authors have shown has a disciplining effect (Griffiths 2013; Boochani et al. 2020).

This uncertainty does, to some degree, also characterise the situation of staff. Despite occupying positions of significant power in relation to detained migrants, they are to a certain extent in a situation of shared vulnerability to the virus inside the detention centres. This was confirmed when the Parliamentary Ombudsman made virtual visits to two different detention centres during the pandemic (Parliamentary Ombudsman Dnr O 23-2020). Their report (18–2020) details how staff in one of the detention centres experienced difficulties maintaining physical distance inside the centre. Staff also reported high numbers of sick leaves during spring 2020. Hence, when it comes to the SMA’s lack of compliance with their own rules and regulations, we wish to underline that this responsibility is only partially the responsibility of (individual) staff. Instead, we wish to draw attention to the institutional and structural disregard for the people incarcerated in migration-related detention, which permit for regulations and recommendations to be disregarded and breached, without anyone being held accountable.

### Indifference to the Health and Well-being of Detained Migrants

“When we ask to see a doctor, it takes a long time before we can see the doctor, and I’m afraid I will get infected with Corona, but unfortunately, nobody cares.”

“Help us, I don’t want to die in here.”

“I’m afraid staff have seen the people who are infected and even the person who has died.”

“I don’t know if I will live or die here. Because every day people get infected and I’m scared.”

“If you think you have COVID-19 they put you in a room by yourself for seven days without taking you to the hospital.”

The above quotes demonstrate the widespread concern and fear among detained migrants over authorities’ lack of care for their health. They also indicate the limited availability of professional medical and psychological support in the detention centres. More than 57% of the respondents reported that they had experienced COVID-19 related symptoms, including coughing, sore throat, headache, and fever. According to the SMA’s incident reports for 2020, four detained people had confirmed COVID-19 infections. In March 2020, one man who fell ill inside detention died from the virus after having finally been admitted to the hospital. Meanwhile, 37% of our respondents reported that they had not been granted adequate access to a nurse or psychologist, even though they had asked for help. 19% stated that they had received psychosocial support once and only 9% had more than one conversation with a psychologist. Half of the respondents in one detention centre claimed that they had not been allowed to meet healthcare personnel despite having asked to see a doctor.

Once more, we hear detained people articulate feeling abandoned and as if ‘nobody cares’ for them. Their experience resonates with observations made in detention centres elsewhere, where detained migrants’ physical and mental vulnerabilities are neglected—and often even amplified—while they are in confinement (Canning 2019; Boochani et al. 2020). The survey responses further demonstrate how detained people experienced fear and resignation due to their limited ability to protect themselves from the virus. The reported lack of availability of professional medical and psychological support indicates a violation of the Communicable Diseases Act (2004:168),^4^ which states that authorities should take measures to protect both non-infected persons and those carrying the disease.

### Destitute and Disregarded: The Condition of People Released from Detention during the Pandemic

The final expression of governance through ignorance that we identify is illustrated by how non-deported migrants were left unhoused and with no recourse to public funds during the ongoing pandemic. The people exposed to such destitution encompassed those released from detention, and people who had been excluded from social welfare provisions following a final expulsion order. Many of these people were already in a difficult health situation, aggravated by recent years’ legal changes (notably the 2016 change in the Law (1994:137) on the reception of asylum seekers and others), which were supposed to increase the incentives for independent return by limiting their access to housing and to social welfare (Lundberg and Kjellbom 2021)*.* However, instead of resulting in more people leaving Sweden, the restrictive legislation has reportedly made many non-deported people destitute (see Lindberg 2020; Lundberg 2020). The pandemic now further aggravates the situation for these people, whose legal claim to remain due to impediments to deportation enforcement remain unrecognised, something that has also been highlighted in a state enquiry in 2017 (SOU 2017:84).

In spring 2020, civil society organisations supporting young migrants living under threat of deportation reported that they had encountered homeless young migrants who showed symptoms of illness. Due to the risk of contagion, the organisations had to turn them away, although they knew that they had nowhere else to go (Inci et al. 2020). Similarly, FARR have been in contact with people who have been released from detention and who do not have access to shelter or social welfare. These people have very limited possibilities to protect themselves and others from the virus—and nowhere to quarantine. While they have formal access to the Swedish healthcare system, many are hesitant to seek help for fear of being reported to police and migration authorities. Yet, calls from civil society actors for the government to enable municipalities to support these groups, for instance, by opening up hostels where they could quarantine, have gone unheard.

These examples demonstrate the sustained political ignorance for the harmful and counterproductive effects of deportation policies. Making non-deported people homeless enables state authorities to effectively ignore or ‘derecord' them (Kalir and van Schendel 2017), although they continue to be present on the territory and face difficult conditions. Pushed out (Sassen 2015) from support systems, from official registries and thereby from politics, these people are rendered ‘superfluous’ to society (Lundberg 2020). The violence of this orchestrated ignorance (Canning 2018) is only becoming more acute during a pandemic, where being pushed out means being deprived of the possibility to protect oneself—and others—from the virus.

## Discussion

This article has departed from the testimonies of people detained on migration-related grounds in Sweden during the spring of 2020 during the first wave of the COVID-19 pandemic and discussed Swedish government authorities’ deficient measures to protect them as a case of *governance through ignorance*. The pandemic has rendered visible and accentuated some of the violent effects of detention and deportation—although the structural and endemic harms of deportation regimes are no less severe in ‘ordinary’ times. The harms of deportation legislation, which operates in what Veitch (2007) has called zones of irresponsibility, before as well as during the ongoing pandemic (see also Esposito et al. 2020a, 2020b) result from active political choices, which contrary to popular assumptions of welfare states like Sweden being proponents of equality, are premised on a racialised hierarchisation of human life and worth. Indeed, the orchestrated ignorance (Canning 2018) of the harmful effects of detention and destitution that directly result from repressive deportation laws are rendered possible through the racialised, gendered identity of the detained and deportable ‘Other’. The intentions or strategies of frontline bureaucrats in the deportation regime are not our main interest here; rather, we have identified how the political ignorance of the lives of racialised detained and deportable people *structures* and *enables* certain legal interpretations and practices. Hence, we understand the ignorance of the ineffectiveness, and harmful consequences, of deportation law as a structured rather than accidental feature of the Swedish deportation regime.

From the survey data and from our previous research and activist engagements, we can identify certain factors that structure the specific legal interpretations that have been revealed in this article, and which illustrate how ignorance operates on the structural, institutional, and micro level. *Structural* ignorance is visible in how the adverse, ineffective, and harmful effects of ever more restrictive deportation policies are systematically overlooked in political and policy discourse. Public ignorance of the rights, well-being and health of deportable people is rooted in a racism that is denied yet embedded in the very foundations of Nordic welfare states and societies (Gilroy 2006), and which is further facilitated through racialised, gendered political and media discourses that portray deportable migrants as a threat to the security and cohesion of society (also see Djampour 2018; Elsrud 2020). On the *institutional* level, i.e. the level of state authorities such as the SMA, ignorance is enabled through the division of responsibilities and tasks between different state agencies, and non-state organisations that are called upon to fill the gaps in the provision of e.g. social welfare to non-deported migrants who are excluded from formal welfare systems (Eule et al. 2019; Jansson-Keshavarz et al. 2021). It is also facilitated through the responsibilisation of people on the move for their own ‘illegality’ (Lundberg 2020), and the silencing of migration law’s violent implications for the individuals and communities affected is allowed (Boon-Kuo 2018). Finally, on the micro-level, i.e. in the everyday relations between personnel and detained people, we identify that ignorance operates through how people experience opacity and significant uncertainty regarding their situation and the political decisions that affect them.

What are the implications of the governance through ignorance? The consequences of ignorance are most palpable at the individual level. People who have been detained during the pandemic express strong anxiety, in some cases a deep fear, that Sweden does not care for their health, rights and well-being. Our respondents report being physically unable to protect themselves and others from the virus inside detention centres and significant uncertainty regarding how far government authorities are willing or able to provide them with adequate protection. In the worst case, they risk dying, and they are acutely aware of this risk. The significant mental and physical harms that detained people are exposed to during the pandemic need to be weighed against the official purpose of migration-related detention: namely, to facilitate deportation enforcement. This is illustrative of the structural ignorance at play, where the harmful effects of deportation are systematically disregarded—even in a time where the real possibilities to deport are basically nonexistent due to the pandemic. In sum, the political and financial economy of deportation (Peutz and De Genova 2010) clearly takes priority over migrants’ health; as one respondent aptly put it, Sweden is ‘doing business’ with their lives.

The study also sheds light on some of the problematic facets of the often-praised Swedish trust in government institutions. In the Swedish institutional context of detention—which as elaborated on above, is located at the geographical and legal margins of the migration control apparatus—there are practically no possibilities for redress. Hence, the Swedish Migration Agency can ignore the government’s guidelines, without any implications and with no self-criticism. The ‘business’ is well protected from transparency and the review functions are toothless, both because of legal-bureaucratic technicalities and due to the generalised trust that Swedish public authorities tend to succeed in what they do, including enforcing deportations. Hence, we find that the perpetration of harm against detained and deportable people is further enabled through a nationalist ideology that includes high trust in ‘the state’ as the guarantor of equal rights, rule of law, and fairness of judicial procedures. This conclusion is in line with prior research, which has highlighted how high levels of trust in government institutions, and belief in equality and social solidarity on a societal level, are key features of Swedish welfare nationalism (Barker 2018). The narratives of inclusive welfarism have further contributed to rendering invisible the discrimination and social exclusion that disproportionately targets non-citizens and racialised citizens, while responsibilising marginalised groups for their own exclusion (Tervonen et al. 2018).

Finally, and crucially, the Swedish migration authorities’ (non)response to the pandemic has rendered visible the racialised, gendered and classed boundaries of the welfare state, which effectively exclude and disregard those ascribed the status ‘deportable migrants’. This dehumanising classification denies them status as rights-bearing subjects. As a result, their lives are not considered as worth protecting, and harms and risks can be ignored which would not have been considered acceptable, had they been citizens. With this article—and the collaborative, activist methodological approach that it builds on—we have attempted to address and challenge the dehumanisation that sustains such hierarchical classifications of human life and worth. Indeed, we believe that ignorance not only affects those who risk expulsion, but has more profound implications for our society, as it enables a gradual erosion of solidarity and equality of rights and protections, including common ideas about what a welfare society is.

The more general and long-term effects of the management of COVID-19 for detained and deportable people—and for migration and deportation politics at large—cannot be predicted at present. Ignoring basic needs and rights, as well as national laws which are intended to protect people from violations and maintain a trust in law and order, as has been done, is in general a dangerous game. Early in the pandemic, we saw alternatives being articulated in countries like Portugal, which—although only temporarily—granted access to social welfare, including healthcare, for everyone present on the territory, regardless of their migration status. Instead of continuing pursuing policy agendas that have proved ineffective and harmful—particularly during a pandemic, but also at other times—governments should consider such programmes for regularisation rather than illegalisation. Meanwhile, we need to keep working to challenge the institutions that sustain racialised hierarchisations of human life and demarcations between ‘us’ and ‘them’, an endeavour that must include and should depart from the concerns and struggles of the people affected by migration enforcement. One of the aims of this article has been to suggest methods and analytical instruments for doing so. COVID-19 is an illustrative example of how the ignorance of detained and deportable people’s lives, which is enabled through the systemic racism that is vehemently denied by Swedish policymakers and bureaucratic authorities, causes direct physical and psychological harms to racialised ‘Others’. What is more, it also dehumanises the society perpetuating these harms. There is, conclusively, an urgent need to challenge harmful deportation laws and policies, which, as this article has shown, are not exceptional but intrinsic to the operation of migration control regimes.

## Footnotes

1 The commission for review of legislation, law enforcement and legal security for people who applied for asylum in Sweden during the period 2015–2017—a collaboration between Linköping University and the Swedish Network of Refugee Support Groups, see (https://liu.se/forskning/asylkommissionen).

2 Respondents could access the online link via computers, which are available in a common computer room. Detained persons are not allowed to use smart phones inside the centres. The survey was tested on two people with experience of seeking asylum, who confirmed that the questions were comprehensible. Survey responses were provided in English and Swedish. The Swedish quotes have been translated by the authors of this article.

3 Aliens Act 2005:716 Chapter 10 § 1-9. See: SFS 2005.716 Utlänningslag. https://www.riksdagen.se/sv/dokumentlagar/dokument/svenskforfattningssamling/utlanningslag-2005716_sfs-2005-716. (Accessed 18 August 2022)

4 Communicable Disease Act 2004:168 SFS 2004:168. Smittskyddslag. https://www.riksdagen.se/sv/dokumentlagar/dokument/svenskforfattningssamling/smittskyddslag-2004168_sfs-2004-168. Accessed 18 August 2022.
